# Tourette Syndrome and Comorbid Neuropsychiatric Conditions

**DOI:** 10.1007/s40474-016-0099-1

**Published:** 2016-11-05

**Authors:** Ashutosh Kumar, William Trescher, Debra Byler

**Affiliations:** Penn State Milton S. Hershey Medical Center, 500 University Drive, Hershey, PA 17033 USA

**Keywords:** Tourette syndrome (TS), Neuropsychiatric comorbidities, Attention deficit hyperactive disorder (ADHD), Obsessive compulsive disorder (OCD), Neurologic complication, Migraine

## Abstract

Tourette syndrome is a neuropsychiatric condition characterized by both motor and phonic tics over a period of at least 1 year with the onset in childhood or adolescence. Apart from the tics, most of the patients with Tourette syndrome have associated neuropsychiatric comorbidities consisting of attention deficit hyperactivity disorder, obsessive compulsive disorder, rage attacks, sleep issues, depression, and migraine. Patients may also have physical complications directly from violent motor tics which can rarely include cervical myelopathy, arterial dissection, and stroke. The purpose of this article is to review the associated neuropsychiatric comorbidities of Tourette syndrome with emphasis on recent research.

## Introduction

Gilles de la Tourette syndrome (TS) is a neuropsychiatric condition characterized by both motor and phonic tics with the onset in childhood or adolescence. First described by French physician Georges Gilles de la Tourette in 1885, it is considered to be as one of the most common childhood movement disorders. As per the Diagnostic and Statistical Manual of Mental Disorders Fifth Edition’s (DSM-V), TS is diagnosed clinically by the presence of multiple motor and one or more phonic tics, lasting at least 1 year with the onset prior to age 18 [1]. TS is commonly diagnosed between 2 and 15 years of age with the mean age of onset is 5–7 years. A tic is a brief stereotypic paroxysmal motor activity or vocalization which is often preceded by a premonitory urge or sensation [2]. “Premonitory urges” are a cardinal feature of tics; however, it is often difficult to elicit this history in younger children. Other characteristics of tics include suppressibility, suggestibility, and distractibility which may mimic “psychogenic movement disorder” and can lead to a wrong diagnosis [3]. Tics can be very variable, with typically a waxing and waning course; however, severity typically peaks during early teenage years. Tics typically start as simple tics and become more complex over the time. Motor tics typically predate vocal tics. Tics typically tend to abate in most patients by late teenage or adult years.

The epidemiology of TS is variable depending on the population studied. In one study, the prevalence of TS in a pooled population was reported to be 0.52 % (with 95 % confidence interval 0.32–0.85). TS more commonly affects males than females (ratio of approximately 4:1) [4]. The prevalence of comorbidities was 85.7 %. Over 70 % of patients had attention deficit hyperactivity disorder or obsessive compulsive disorder, and in around 30 % of the patients, mood, anxiety, and disruptive behavior were reported [5]. There are rare but serious cases of associated cervical myelopathy, stroke, and dissection secondary to violent motor tics [6]. There have also been reports of increased prevalence of migraine in patients with TS [7].

The exact pathophysiology of TS is unclear at this point. Various hypotheses have been postulated. It is considered to be a complex disorder, thought to be the effect of environment (infection, autoimmunity as well as perinatal problems) in a genetically predisposed individual. In one study, TS patients were found to have decreased gray matter thickness in the pre- and post-central and internal, inferior, and superior frontal sulci that correlated with clinical severity of the tics [8]. Various other studies suggested that TS is due to dysfunction in the cortico-striato-thalamo-cortical circuit [9–11]. The cause of the dysfunction in the cortico-basal ganglia structures is not clearly defined; however, abnormalities of the gamma-aminobutyric acid (GABA) pathway are postulated to cause the disinhibition seen in TS patients [12]. To simplify, the basal ganglia (caudate, striatum, and globus pallidus) is involved in the control of voluntary movements and abnormalities affecting these areas result in a variety of movement disorders including TS.

The genetics of TS is still under investigation. TS is postulated to be a familial disorder, likely with bilinear inheritance (both parents affected) [13]. One study noted an overall heritability of 0.77 in tic disorders, greatest for first degree relatives [14]. Despite advancement in genetic studies, a single causative gene has not been found [15]. Studies have found an association between TS and histamine decarboxylase (HDC) gene [16, 17]. L-histamine decarboxylase, encoded by HDC gene, is the rate limiting enzyme in histamine synthesis. In addition, there may be some association of various single nucleotide polymorphisms (SNPs) noted by genome wide association studies (GWAS) involving proteins encoding axon guidance and outgrowth in the developing striatum [18].

In this review, we discuss the various neuropsychiatric comorbidities seen in TS patients. These comorbid conditions are often the main source of impairment in the TS patients and compromise overall well-being much more than the tic severity. Because of this high rate of comorbidity, any patient presenting with tics should have a thorough investigation for associated comorbidities.

### Attention Deficit Hyperactivity Disorder

Attention deficit hyperactivity disorder (ADHD) is a complex neurobiological disorder, characterize by symptoms of inattention and hyperactivity/impulsivity [19]. It is the most common comorbidity in the TS patients, ranging between 60 and 80 % depending on the type of patient population studied [20, 21]. ADHD symptoms precede the onset of tics by 2–3 years and begin around the age of 3–5 years. The hyperactive subtype is predominant in the younger patients, whereas inattentive type is more common in adult patients, who also have higher risk of substance misuse and aggressive behavior [22, 23]. The pathogenesis of ADHD in TS patients is complex and includes environmental, genetic, and neurobiological factors [24, 25]. Abnormal level of neurotransmitters (dopamine and glutamate) along with loss of normal globus pallidus symmetry is reported in both [26]. The exact genetic mechanism is yet to be elucidated; however, multiple genes have been postulated in the pathogenesis of ADHD in TS patients, which includes catechol-O-methyltransferase (COMT), dopamine receptor D2 (DRD2), monoamine oxidase A (MAOA), solute carrier family 6, member 4 (SLC6A4), myelin-associated oligodendrocyte basic protein (MOBP), dopamine receptor D1 (DRD1), and fatty acid desaturase 2 (FASD2) [27, 28]. The most important neurobiological network involved in pathogenesis is the cortico-striato-thalamo-cortical circuit, which is implicated in tic generation and in impulsivity as seen in ADHD patients [26, 29]. Studies have suggested an involvement of the basal ganglia circuit, especially hypo functioning of the direct pathway resulting in abnormal behavior causing problems with sustaining attention and impulsive action [29]. The treatment of ADHD in TS patients is multimodal and includes a combination of psychoeducation to the patient and family, cognitive behavioral therapy, and pharmacological interventions. Three classes of medications are commonly used for the treatment of ADHD in TS patients, including stimulants (methylphenidate, dextroamphetamine), alpha-agonists (clonidine, guanfacine), and norepinephrine reuptake inhibitors (atomoxetine). In the past, there has been concern regarding the effect of stimulant treatment on the exacerbation of tics. However, recent studies have shown beneficial effect of stimulants in TS patients with comorbid ADHD symptoms [30]. Alpha-2 agonist (clonidine, guanfacine) are considered to be the first line treatment given proven efficacy for both tics and ADHD symptoms. Stimulants are suggested to be the second line of treatment, especially useful in children requiring immediate relief of ADHD symptoms, while it has a very little evidence to support for the treatment of the tics, and in-fact, there is concern about precipitating tics in some patients. More recently, the partial dopamine agonist (aripiprazole) has been recommended for the treatment if TS with mild ADHD symptoms [31]. A non-invasive modality known as repetitive transcranial magnetic stimulation (rTMS), in which changing magnetic fields stimulate targeted areas of the brain, shown to alter cortical activity, has been tried for the treatment of ADHD and TS, with conflicting results and further investigations have been suggested [32, 33].

### Obsessive Compulsive Disorder

Obsessive compulsive disorder (OCD) is defined by the presence of obsessions (intrusive thoughts) and compulsions (repetitive behavior) leading to adaptive malfunctioning and emotional maladjustment [34]. The prevalence of OCD ranges from 11 to 80 % of patients with TS [35]. The exact etiology of OCD in TS patients is unclear; however, studies have suggested an involvement of the basal ganglia circuit, especially disruption of the indirect pathway resulting in repetitive behaviors and thoughts. [29]. OCD symptoms have been reported to arise any time during the course of TS. Interestingly, compulsive symptoms were more common than obsessions if OCD is the presenting symptom in TS patients [36]. The presentation of OCD symptoms in TS patients can be different than the typical presentation of patients with primary OCD. For example, TS patients are reported to have greater rates of symmetric obsession, obsessional counting (arithmomania), and “just right” perception, whereas primary OCD patients reports higher prevalence of cleaning rituals, compulsive washing, and fears of contaminations [55]. Treatment of OCD in TS patients is multimodal including cognitive behavioral therapy and pharmacotherapy. Recommended pharmacotherapy can include atypical antipsychotics (risperidone) combined with a selective serotonin reuptake inhibitor (SSRI) [37]. Thalamic deep brain stimulation has been suggested in selected cases of refractory TS with comorbid OCD; however, existing data is inconclusive, and the procedure remains experimental [56].

### Rage Attacks

Twenty-five to seventy-five percent of TS patients have episodic behavioral outburst and anger issues [38]. There has been a significantly increased prevalence of impulse control disorder (especially intermittent explosive disorder) in adult TS patients. Atypical antipsychotic (risperidone) have been reported to be effective in reducing the intensity and frequency of the rage attacks [39].

### Depression

Thirteen to seventy-six percent of patients with TS reported to have depressive symptoms [40]. The mechanism of development of depression is multifactorial including a psychological reaction to potentially disabling condition and associated social stigma (many children experience bullying, teasing, and receive derogatory nicknames) and multiple neurotransmitter abnormalities and potential side effect of medications commonly used to treat tics (especially neuroleptics). Selective serotonin reuptake inhibitors and tricyclic antidepressants have been reported to be effective in the treatment of depression in TS patients [41].

### Sleep Issues

Twelve to sixty-two percent of patients with TS have been reported to have various sleep problems including REM (nightmares), NREM sleep disorders (night terrors, somnambulism), trouble initiating sleep, and restlessness [42]. Clonidine and melatonin have been reported to be effective in these patients [43].

### Migraine

In a study done by Kwak et al., 25 % of TS patients satisfied the diagnostic criteria of migraine compared to 10–13 % of general adult population with *p* value of <.001 and concluded that migraine is an another comorbidity in TS patients [7]. There was a very similar observation made by Barabas et al., who reported that 27 % of patients with TS met the diagnostic criteria for migraine headache [44]. The mechanism is unclear; however, a defect in the serotonin metabolism has been postulated [45].

### Learning Disabilities

In a large study (*n* = 5450), 23 % of patients with TS had associated learning disabilities [46], whereas older studies with small patient population have reported even higher prevalence (50 %) of learning disabilities (LD) [47]. Learning disability was more common in males than females (*p* < .001). Patients with learning disability also had increased rates of other comorbid conditions, most notably ADHD (80 % of patients with TS and LD). This study suggested that ADHD could be a causal factor or a confounder for the diagnosis of LD as only 11 % of the TS children without ADHD had LD [46]. Learning disabilities in math and spelling are the most frequent. The patients also have increased rate of reading disorder. In most patients, LD is present by the time TS is diagnosed, and therefore, patients need to be evaluated by neuropsychological testing so that they can receive appropriate educational accommodations.

### Rare Neurological Complications

TS can rarely be associated with more severe complications which include cervical myelopathy, cervical disk herniation, compressive neuropathy, stroke, and arterial dissection [48–53]. Patterson et al. recently published four cases of cervical myelopathy and arterial dissection, leading to spinal cord injury and stroke secondary to violent motor tics involving the neck in patients with TS. They suggested aggressive treatment of violent tics to prevent these dreaded complications and recommended using cervical collar for stabilization and prevention of initial injury and to halt progression of existing injury [6]. Termine et al. suggested treatment with botulinum toxin to prevent these severe complications [54]. In these cases, the comorbidities are complications of the actual physical tics rather than neuropsychiatric complications arising from a parallel, secondary, or associated neurochemical phenomenon.

### Approach to the Patient with a Tic Disorder

Patients with tics disorders are often focused on the involuntary movements or noises that bring them to medical attention. The diagnosis of a tic disorder is often made without much difficulty, and when the criteria for symptom complexity and duration are met, the diagnosis of Tourette syndrome is applied. However, a thorough knowledge of the comorbidities often associated is essential in the evaluation and longitudinal care of these patients. Patients and families may be unaware or minimize neuropsychiatric comorbidities, and specific inquiry and investigation are often quite rewarding and guides treatment. The conditions which are causing the most distress and alteration in life quality are given priority in the treatment plan, and very often, these are not the motor tics which may have brought the patient initially to medical attention.

## Conclusion

Tourette syndrome is a complex neuropsychiatric disorder with tics (both motor and phonic), and a variety of neuropsychiatric comorbidities more commonly includes ADHD, OCD, impulse control disorder, rage attacks, sleep issues, depression, migraine and rarely cervical myelopathy, stroke, and dissection due to violent motor tics. The pathophysiology relating to comorbidities in TS is unclear, however thought to be at least in part related to dysfunction in the cortico-striato-thalamo-cortical circuit. Investigation and treatment of comorbid conditions are an essential part of the treatment plan for all patients with TS. Practitioners should also be aware about the rare but severe neurological complications in these patients and consider treating tics aggressively.
